# Non-Thyroidal Illness in Chronic Renal Failure: Triiodothyronine Levels and Modulation of Extra-Cellular Superoxide Dismutase (ec-SOD)

**DOI:** 10.3390/antiox13010126

**Published:** 2024-01-20

**Authors:** Antonio Mancini, Andrea Silvestrini, Fabio Marcheggiani, Emmanuele Capobianco, Sonia Silvestri, Erminia Lembo, Patrick Orlando, Flavia Beccia, Nicola Nicolotti, Nicola Panocchia, Luca Tiano

**Affiliations:** 1 Dipartimento di Medicina e Chirurgia Traslazionale, Università Cattolica del Sacro Cuore, Largo Francesco Vito, 1, 00168 Rome, Italy; 2 Dipartimento di Scienze Biotecnologiche di Base, Cliniche Intensivologiche e Perioperatorie, Università Cattolica del Sacro Cuore, 00168 Rome, Italy; 3 Department of Life and Environmental Sciences, Polytechnic University of Marche, Via Brecce Bianche, 60131 Ancona, Italy; 4 Section of Hygiene, University Department of Life Sciences and Public Health, Università Cattolica del Sacro Cuore, Largo Francesco Vito, 1, 00168 Rome, Italy

**Keywords:** oxidative stress, ec-SOD, low fT3 syndrome, total antioxidant capacity, haemodialysis, chronic kidney diseases

## Abstract

Oxidative stress (OS) is implicated in several chronic diseases. Extra-cellular superoxide dismutase (ec-SOD) catalyses the dismutation of superoxide anions with a protective role in endothelial cells. In chronic kidney disease (CKD), OS and thyroid dysfunction (low fT3 syndrome) are frequently present, but their relationship has not yet been investigated. This cohort study evaluated ec-SOD activity in CKD patients during haemodialysis, divided into “acute haemodialytic patients” (AH, 1–3 months of treatment) and “chronic haemodialytic patients” (CH, treated for a longer period). We also evaluated plasmatic total antioxidant capacity (TAC) and its relationships with thyroid hormones. Two basal samples (“basal 1”, obtained 3 days after the last dialysis; and “basal 2”, obtained 2 days after the last dialysis) were collected. On the same day of basal 2, a sample was collected 5 and 10 min after the standard heparin dose and at the end of the procedure. The ec-SOD values were significantly higher in CH vs. AH in all determinations. Moreover, the same patients had lower TAC values. When the CH patients were divided into two subgroups according to fT3 levels (normal or low), we found significantly lower ec-SOD values in the group with low fT3 in the basal, 5, and 10 min samples. A significant correlation was also observed between fT3 and ec-SOD in the basal 1 samples. These data, confirming OS and low fT3 syndrome in patients with CKD, suggest that low fT3 concentrations can influence ec-SOD activity and could therefore potentially contribute to endothelial oxidative damage in these patients.

## 1. Introduction

Non-thyroidal-illness syndrome (NTIS) is a condition present in acute and chronic illnesses, in the absence of thyroid disease, and is considered an adaptive response rather than real hypothyroidism. The most common picture is low deiodination of free thyroxine (fT4), leading to reduced circulating levels of free triiodothyronine (fT3), which leads to “low fT3 syndrome”. However, in prolonged illness, low fT4 secretion is also involved [1]. Originally described and largely debated in 90’ [2,3], some open questions remain unresolved after more than three decades [4]. In acute conditions, this picture can be observed after starvation or critical illness, such as sepsis or major surgery, while low fT3 is commonly observed in chronic kidney and liver diseases, heart failure, and chronic inflammatory diseases [1]. A key role appears to be played by increased cytokines levels, but other mechanisms can be involved, such as alterations in plasma thyroid hormones transporter, clearance, and modifications of membrane transporters [5]. It is also claimed that selenium has a role, as this element is implicated in the mechanism of enzymatic deiodination [6]. Different regulation of deiodinases 2 and 3 (DIO2 and DIO3) modulates fT3 intracellular levels with subtle tuning to compensate for systemic TH unbalance [7]. We have previously described as thyroid hormone influence antioxidant systems, and their evaluation in chronic disease can represent an index of tissue effects of these hormones [8]. 

Thyroid hormone replacement therapy is usually not prescribed in this particular clinical context, but this topic is still under debate [9]. Indeed, a tissue-specific response has been described: some studies have suggested that, at a cellular level, reduced fT3 bioavailability can be hazardous and represents a “maladaptive” rather than “adaptive” response. Intracellular oxidative stress has been hypothesised to be one of these consequences [8].

Oxidative stress (OS) may worsen the NTIS condition in a vicious cycle related to alterations in deiodinase and the adverse impacts of low fT3 on antioxidant levels or activity [10]. Consequently, chronic inflammation and OS reciprocally affect each other, leading to more severe clinical progression of these conditions [8]. Based on both experimental and clinical studies, the levels of thyroid hormone (TH) in NTIS do not necessarily reflect their low concentrations in the bloodstream and their levels within cells. Furthermore, reports suggest that thyroid hormone transport, receptor binding, and hormone metabolism are specific to tissues [1].

Oxidative stress is often reported in chronic kidney disease (CKD) [11]. In our previous study we reported that in CKD total antioxidant capacity (TAC) significantly correlated with the irisin levels [12], a peptide involved in the browning of white adipose tissue, protecting it from insulin resistance and increasing energy expenditure [13,14]. Among the consequences of OS, endothelial dysfunction is related to cardiovascular complications reported in CKD [15].

Superoxide dismutases (SODs) are an important class of enzymes that protect against the formation of oxidants [16]. Extra-cellular superoxide dismutase (ec-SOD), also known as SOD3A, is a unique extracellular antioxidant enzyme with a key function in the vasculature in the prevention of oxidant-mediated tissue damage. The ec-SOD was originally discovered in 1982 by Marklund [17] and is different from the mitochondrial MnSOD (SOD2) or cytosolic CuZnSOD (SOD1) isoforms; although closer to the latter, it contains a heparin-binding domain that allows it to bind to the glycosaminoglycans of extracellular matrix. In particular, in the arterial wall, it constitutes the primary endogenous defence against vascular superoxide anions and is critical for endothelial health in the protection of nitric oxide. The endothelium-bound ec-SOD can be assessed in patients by measuring the increase in plasma superoxide scavenging activity triggered by an injection of heparin in the patient, which leads to transient competition with heparan sulphate groups in the extracellular matrix and a consequent increase in ec-SOD plasma levels. 

Although its significance is acknowledged, limited information exists on ec-SOD in patients undergoing haemodialysis [18,19]. This lack of comprehensive study underscores the necessity for further research in this area. Moreover, its relationships with thyroid hormones has not yet been investigated. Haemodialytic patients represent an interesting model to evaluate ec-SOD, as an acute release of ec-SOD in the blood is induced by heparin administration before starting the dialysis procedure. 

Therefore, this observational cohort study was designed to evaluate, as the primary endpoint, the levels ec-SOD in two models of NTIS: (1) patients with chronic kidney disease undergoing haemodialytic treatment that started a short period ago (“acute haemodialytic patients”, AH); and (2) patients who have been undergoing haemodialysis for a longer time period (“chronic haemodialytic patients”, CH). This observational study has as its secondary endpoint the measurement of ec-SOD activity in correlation with thyroid hormones, to establish the possible effects of NTIS in the modulation of antioxidant systems.

## 2. Materials and Methods

### 2.1. Patients Enrolments

Patients with end-stage renal disease (ERDS) on maintenance haemodialysis were considered eligible for inclusion. Patients with ESRD undergoing thrice-weekly maintenance haemodialysis at the University Hospital “Agostino Gemelli” were screened. Exclusion criteria were as follows: advanced heart failure (according to the criteria of the European Society of Cardiology); diagnosis of dementia based on DSM-IV criteria; history of alcohol or substance abuse; a previous diagnosis of a psychotic disorder; and clinical instability requiring hospital admission. Seventy-one patients were assessed for participate in this study. Five did not given their consent to participate, while ten were excluded according to the exclusion criteria above. The remaining 56 were screened, and 46 were finally included in the study (75% response rate). Forty-two were affected by hypertension, and were treated as appropriate with beta-blockers, calcium antagonists, ACE-inhibitors, and sartans (Angiotensin II receptor blockers). Fourteen had a diagnosis of diabetes, but none exhibited poor control of the disease (indicated by glycosylated haemoglobin >7%). None of the enrolled patients were prescribed corticosteroids or dopamine. 

Two groups were defined according to the start of haemodialysis administration, i.e., if it starts in the first period of treatment or continues for a longer time:

- Acute Haemodialysis (AH) group: 1–3 months of treatment
- Chronic Haemodialysis (CH) group: 12–180 months of treatment

All patients were given an explanation of the purposes and nature of the study, and written informed consent was obtained. The study protocol had been approved by the Institutional Board of the School of Medicine, Catholic University (Prot. n. 15291/13). All patients received 4 h bicarbonate haemodialysis three times a week, according to the schedule employed in the haemodialysis unit. The blood flow ranged from 250 to 300 mL/min, with a dialysate rate flow of 500 mL/min. Patients in group AH underwent haemodialysis via venous central catheter (CVC), and patients in Group CH were dialysed via CVC or arteriovenous fistula. All patients were treated with high-permeability membranes. Membranes were not reused. The inter-dialytic weight gain (IDWG) and pre-dialysis systolic blood pressure of 10 consecutive haemodialysis sessions (the same used to record IDH) were recorded, and mean and median values were calculated [20].

### 2.2. Sample Collection 

Between 08.00 and 09.00 a.m., before starting haemodialysis treatment, when fasting, a venous blood draw was performed. A blood sample was collected, via the arteriovenous fistula (in CH) or central venous catheter (both in AH and CH), immediately during the “short interval” (48 h after the last dialysis, “basal 2”) or “extended interval” (72 h after the last dialysis, “basal 1”) to evaluate differences in the basal levels of ec-SOD linked to the duration of the intervals between dialytic procedures. On the day of a short interval, we also collected samples at 5 and 10 min after heparin administration preceding the starting of haemodialysis, and at the end of the same, according to the dynamic response of ec-SOD to heparin as previously reported [21]. All samples were collected in a test tube containing heparin as an anticoagulant and immediately centrifuged (4 °C at 2500× *g* for 10 min) were subsequently aliquoted in 2 mL and stored at −80 °C until assayed.

### 2.3. Hormonal and Metabolic Parameters

We also collected an aliquot for the determination of the following metabolic and hormonal parameters: glucose, creatinine, uric acid, total cholesterol, LDL-C, HDL-C, albumin, C-reactive protein (CRP), transaminases, fT3, fT4, and TSH.

Plasma concentrations of glucose, creatinine, uric acid, total cholesterol, LDL-cholesterol, HDL-cholesterol, albumin, CRP, and transaminases were measured using enzymatic assays by an Olympus AU2700 chemistry analyser (Olympus America Inc., Center Valley, PA, USA). The intra- and inter-assay coefficients of variation (CV) for total cholesterol and uric acid were <1.5% and <2.5%, respectively. The intra- and inter-assay CV for HDL-cholesterol were <2.5% and <3.0%, respectively.

Serum concentrations of TSH, fT3, and fT4 were measured using immunochemiluminometric assays on a Roche Modular E170 analyser (Roche Diagnostics, Indianapolis, IN, USA). The intra-assay and inter-assay CV for all hormones were, respectively, <5.0% and <7.0%. The normal fT3 range in our laboratory measurements was 2.5–3.2 pg/mL. Thus, low fT3 levels were defined as under 2.5 pg/mL. Moreover, a “severe low fT3” was further defined as an fT3 level under the median of the low fT3 population.

### 2.4. Oxidative Parameters

Total antioxidant capacity (TAC) was evaluated using the method of Rice-Evans, modified in our laboratory as described below [22,23]. This assay is based on inhibition, determined by antioxidants, of the absorbance of the radical cation 2,2’-azinobis (3-ethylbenzothiazoline-6 sulfonate) (ABTS^●+^) formed by interaction between ABTS (150 μM) and ferrylmyoglobin radical species, generated by activation of metmyoglobin (2.5 μM) with H_2_O_2_ (75 μM). Aliquots of the frozen plasma were thawed at room temperature, and 10 μL of the samples was tested immediately. The manual procedure was used with only minor modifications, as previously described [23]. The reaction was started directly in the cuvette by the addition of H_2_O_2_ after 1 min of equilibration of all other reagents and followed for 10 min, monitoring at 734 nm. The presence of chain-breaking antioxidants induces a lag time (the “Lag phase”) in the accumulation of ABTS^●+^ proportional to the concentration of the antioxidants and expressed as the length of such a Lag phase (in seconds). This assay mainly measures non-protein and non-enzymatic antioxidants that are primarily extracellular chain-breaking antioxidants, such as ascorbate, urate, and glutathione. Trolox, a water-soluble tocopherol analogue, was used as a reference standard. Absorbance was measured using a Hewlett-Packard 8450A UV/Vis spectrophotometer (Palo Alto, CA, USA) equipped with a cuvette stirring apparatus and a constant temperature cell holder. 

The ec-SOD activity was measured using a modified nitrite method [21]. Briefly, superoxides generated by hypoxanthine and xanthine oxidase were changed by hydroxylamine to nitrite anions, which were measured spectrophotometrically at 550 nm via the use of a chromogen. The amount of ec-SOD required to inhibit 50% nitrite anion generation was defined as 1 U of ec-SOD activity.

### 2.5. Statistical Analysis

Descriptive analyses were performed using frequencies and percentages for categorical data, and mean and standard deviation (SD) for continuous data. Skewness and kurtosis were used to investigate the distribution of the collected quantitative data and the Shapiro–Wilk test was used to investigate if data had a normal distribution. To investigate differences between groups and within the groups for the ec-SOD activity values after heparin infusion (at 5 and 10 min and at the end of treatment) vs. basal values (basal 2 short), statistical tests were deployed, namely Student’s *t*-test or the Wilcoxon rank-sum test (Mann–Whitney) or sign-rank test, for unpaired and paired data, respectively, for quantitative data according to the normality of the distribution. The Fisher correction was applied to investigate differences between groups and within the groups for the ec-SOD activity values after heparin infusion (at 5 and 10 min, and at the end of treatment) compared to basal values (basal 2 short). In the CH group, ec-SOD activities were compared with the levels of fT3. Paired or unpaired tests were deployed accordingly. Pearson bivariate correlations were performed to check for multi-collinearity between fT3 levels and ec-SOD activities. Statistical significance was set at *p* < 0.05. The statistical analysis was performed using the STATA 17.0 software package (Stata Corporation, College Station, TX, USA).

## 3. Results

A total of 56 patients were screened and 46 were included in the study. Overall, 30 (65.22%) were male, and the mean age was 68.27 years (SD 14.42), with an overall haemodialysis time of 55.25 months (SD 68.9). According to the treatment duration, 10 patients were allocated to the AH group and 36 to the CH group.

Metabolic parameters in the AH and CH groups are described and compared in Table 1.

Hormonal parameters and antioxidants activity in both groups are reported and compared in Table 2.

All parameters were within normal range according to our clinical biochemistry parameters, except for creatinine, as expected, with significantly higher values in the CH patients. Albumin and GPT (Glutamic-Pyruvic Transaminase) values were higher in the AH group, with a statistically significant difference from the CH group. Hormonal parameters reported in Table 2 show significant variations between chronic and acute patients, with significantly higher levels of ec-SOD and LAG lower levels in the chronic group.

**Figure 1:**
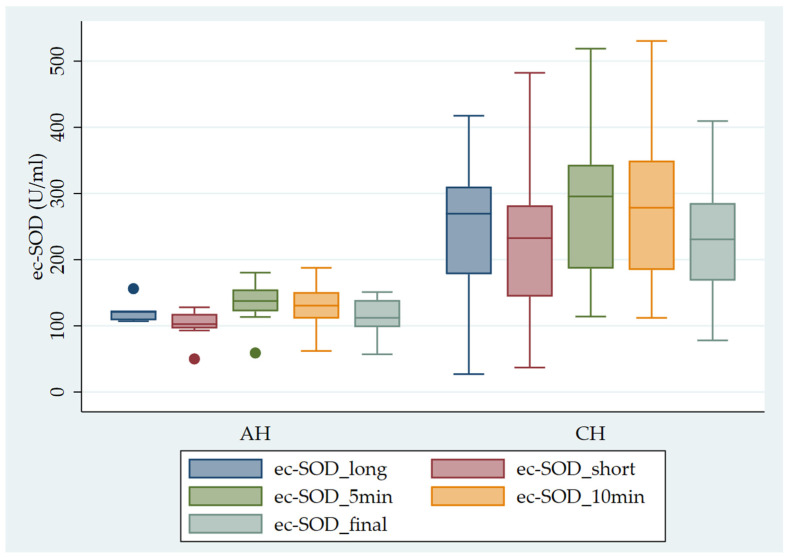
Pattern of ec-SOD (U/mL) release after heparin infusion in AH vs. CH patients. Error bar indicates the standard error. ec-SOD_long = basal fasting sample collected after a long interval; ec-SOD_short = basal fasting sample collected after a short period; ec-SOD_5min = activity values after heparin infusion at 5 min; ec-SOD_10min = activity values after heparin infusion at 10 min; ec-SOD_final = activity values at the end of treatment.

Figure 1 shows the pattern of ec-SOD release after heparin infusion in AH vs. CH patients.

**Figure 2:**
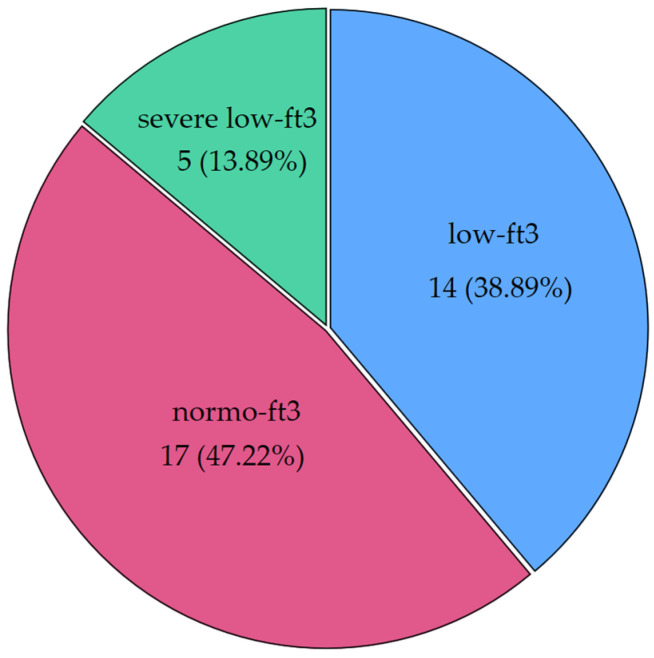
Distribution of low fT3 syndrome in our cohort of chronic patients (CH group) (severe low fT3 was defined as a condition with fT3 levels below the median of the whole low fT3 population). Severe low fT3 < 1.8 pg/mL; low fT3 between 1.8 pg/mL and 2.4 pg/mL; normal fT3 ≥ 2.5 pg/mL. Below the label, frequencies and percentages are displayed.

Interesting patterns were observed when CH patients were classified according to fT3 levels. Nineteen patients (52.78%) had low levels of fT3, and a quarter of those (26.32%) had severe NTIS (<1.8 pg/mL, the median of low fT3 values). Figure 2 shows the distribution of fT3 categories (severe NTIS: <1.8 pg/mL; low fT3 between 1.8 pg/mL and 2.4 pg/mL; normal fT3 ≥ 2.5 pg/mL).

Figure 2 shows ec-SOD levels after heparin infusion in CH patients divided according to the presence of a low fT3 condition.

Basal ec-SOD activity differed between the low and normal fT3 groups only for the basal 1 (long) measurement, but the increase after heparin was significantly correlated with fT3 levels. In Table 3, the different patterns of ec-SOD release after heparin are described and compared in the two subgroups.

**Figure 3:**
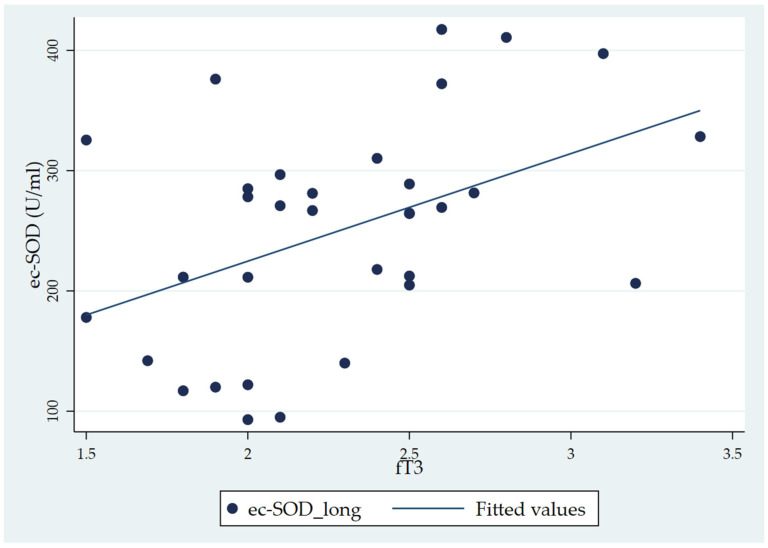
Scatter plot showing the positive correlation between fT3 and ec-SOD (U/mL) activity (basal 1 sample) in chronic patients (the CH group).

When considering the total fT3 distribution, the Pearson correlation analysis showed a significant correlation of fT3 with ec-SOD basal 1 (long) (r = 0.443, *p* = 0.01) (Table 4). In the correlation matrix, while ec-SOD measures at baseline, five, and ten minutes and at the end are correlated with each other (as expressed by *p*-values < 0.001), the only significant correlation with the fT3 level is that of ec-SOD at basal 1 (long). The scatterplot of the significant correlation between fT3 and ec-SOD at basal 1, along with the fitted model, is represented in Figure 3.

In the AH group, comparing changes in ec-SOD levels over time until the end of treatment, there was an average increase of 26.36% (SD 10.3%) at 5 min, 21.85% (SD 10.09%) at 10 min, and 8.80% (SD 21.45%) at the end. In the CH group, the increases from baseline were 28.01% (SD 34.77%) at 5 min, 26.50% (SD 34.63%) at 10 min, and 4.24% (SD 31.24%) at the end. Table 5 shows the crude increase and statistical difference by group from baseline values (baseline 2 short). We also performed the same analysis on the low-/normal-fT3 level subgroups of the CH group.

The intragroup comparison highlighted a significant increase in the ec-SOD activities at 5 (*p* = 0.004) and 10 min (*p* = 0.004) after injection in the AH group. Similar results were obtained for the CH group (*p* < 0.001 at both 5 and 10 min versus basal ec-SOD activities) (Table 5).

The increase observed in fT3 levels in the CH group was statistically significant at 5 and 10 min after injection. In the low fT3 subgroup, the *p* values were <0.001. In the normal fT3 subgroup, the *p*-values were 0.002 and 0.001 for both times, respectively.

Comparison of ec-SOD levels at the end of haemodialysis versus baseline was not significant in each group or subgroup analysed.

**Figure 4:**
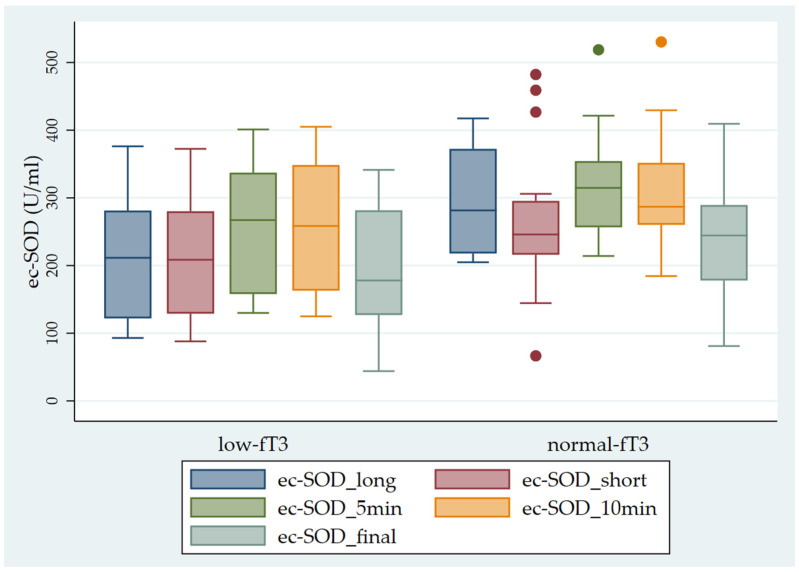
Pattern of ec-SOD (U/mL) release before and after heparin infusion in the two sub-groups of patients studied (low fT3 and normal fT3). The error bars indicate the standard error. ec-SOD_long = basal fasting sample collected after a long interval; ec-SOD_short = basal fasting sample collected after a short period; ec-SOD_5min = activity values after heparin infusion at 5 min; ec-SOD_10min = activity values after heparin infusion at 10 min; ec-SOD_final = activity values at the end of treatment.

Finally, we observed significantly higher ec-SOD values in the sub-groups with normal fT3 levels in comparison with the sub-groups with low fT3 levels (Figure 4).

## 4. Discussion

Non-Thyroidal Illness Syndrome (NTIS) is an adaptive condition in response to numerous acute and chronic diseases, and it is intricately linked to the prognosis of the latter. The underlying mechanisms are complex, primarily involving the deregulation of deiodinases. However, it is essential to note that other factors can also play a role, and their influence may vary depending on the specific tissues involved [1]. In mice, TH expression is decreased in sepsis and acute inflammation, but does not appear to be affected in chronic inflammation. The same scenario is observed in humans, as the expression of monocarboxylate transporter 8 (MCT8) is lower following acute surgical stress than in prolonged illness; however, a compensatory increase in MCT8 has been described in chronic diseases in rabbits [24]. We have previously described the relationship between inflammation and OS including thyroid involvement in cardio-pulmonary diseases [25].

Several studies have reported on the occurrence of OS in CKD and in patients undergoing haemodialytic treatments. Lower enzymatic antioxidants (e.g., glutathione peroxidase, SOD) and higher MDA levels in comparison with controls have been described in this patient group [26]. Interestingly, a correlation with selenium deficiency has been evidenced, and supplementation of this element increased SOD and CAT activities [27]. Moreover, an association between hypoalbuminaemia, biomarkers of inflammation and oxidative stress has been reported [28]. The role of nutrition in CKD has also been emphasized [29].

Concerning ec-SOD, previous research has revealed that patients undergoing haemodialysis have heightened susceptibility to endothelial cell injury, as evidenced by their elevated levels of ec-SOD [30]. Maehata et al. [31] reported an increase in ec-SOD levels in diabetic-nephrotic subjects, particularly notable within the subgroup exhibiting macroalbuminuria. The same researchers evaluated ec-SOD levels with an ELISA method in patients undergoing haemodialysis, describing a correlation between the enzyme, FFA, and LPL, underlying the role of insulin resistance. The same study reported minimal differences between pre- and post-dialysis procedure values, with the latest data contrasting with other findings that demonstrated a notable increase in post-dialytic values [32]. Our preliminary data suggest that low fT3 levels can negatively influence endothelial antioxidant defences. The relationship of this with cardiovascular complications and prognostic usefulness remains to be established.

In contrast with the other SOD isoforms, ec-SOD is the predominant isozyme in extracellular fluids, in particular in plasma. It is a tetrameric glycoprotein (MW 135 kDa) containing one copper and one zinc atom per subunit, which are required for enzymatic activity. The location of the ec-SOD gene in humans is chromosome 4q21 and it shows a 60% homology with CuZn-SOD, but minimal homology with Mn-SOD [33]. The human mRNA for the enzyme is highly expressed in the heart, placenta, pancreas, and lung, and at lower levels in the kidney, skeletal muscle, and liver [33].

The primary location of ec-SOD in tissues is in the extra-cellular matrix and on cell surfaces, where its concentration is 20 times higher than in plasma. The association of ec-SOD with tissue is accomplished by a heparin-binding domain, which recognises heparan-sulphate proteoglycans on the cell surface and in the matrix. Intravenous injections of heparin in humans and other species leads to an immediate increase in plasma ec-SOD content [30], allowing the in vivo determination of endothelium-bound ec-SOD in humans [34]. 

The physiopathological role of ec-SOD has been examined in vascular-related diseases, atherosclerosis, hypertension, diabetes, ischaemia-reperfusion injury, lung disease, various inflammatory conditions, and neurological diseases [33]. The administration of the lipid antioxidant Coenzyme Q10 can induce an improvement in endothelium-bound ec-SOD, presumably by counteracting nitric oxide oxidation [21].

Few papers have reported on the role of ec-SOD in kidney disease. In a model of Adriamycin-induced nephropathy (ADR) characterised by proteinuria and kidney failure, it has been shown that ec-SOD decreased during the illness, with a concurrent increase in NADPH oxidase and oxidative stress index [35]. Interestingly, ec-SOD null mice, with non-proteinuric kidney injury induced by unilateral ureteral obstruction, did not differ from wild-type animals when sensitised to ADR injury: both exhibited augmented NADPH (reduced nicotinamide adenine dinucleotide phosphate) and β-catenin levels. Therefore, it was hypothesised that ec-SOD has a protective role against proteinuria and the progression of the disease. In the same study, human biopsies of chronic kidney disease also showed a similar reduction in ec-SOD levels [35].

A protective role in humans has also been suggested in patients receiving haemodialytic treatment by Yamada [36], who found a single-base substitution of the ec-SOD gene which caused a decrease in its binding capability to endothelial cells and therefore an increased in serum concentration. The percentage of patients harbouring this mutation rapidly decreased in the group of patients with diabetes mellitus; a potential prognostic use was therefore suggested based on patients who died from ischemic heart disease or cerebrovascular accidents.

To the best of our knowledge, we have provided, for the first time, evidence of a relationship between the ec-SOD activity and thyroid hormones in haemodialytic patients, suggesting that low fT3 concentrations can influence ec-SOD levels. According to other chronic conditions, the data seem to point towards real hypothyroidism at the tissue level. Hence, this research could contribute novel insights into the tissue repercussions of NTIS. While the link between low fT3 levels in chronic kidney disease (CKD) and increased mortality is well-established [37], we also propose that NTIS exacerbates antioxidant defences. Indeed, beyond their prognostic significance, fT3 levels may also play a pathogenetic role. However, this study has some limitations which should be discussed. This includes the number of patients and the lack of a control group, due to the difficulty of administering heparin in healthy subjects, and the heterogeneity of our cohort and duration of haemodialytic treatment, even if heterogeneity of patients is a common characteristic of this type of study [8,38].

Further study is required to better understand our observations, that could form the basis for a longitudinal study to evaluate the impact of low fT3 on cardiovascular risk, and for a treatment study, as the topic of usefulness of therapy is still under debate. Moreover, studies at the molecular level can give insight into the tissue effects of NTIS; attention should be directed to nuclear receptor expression, extending previous observations [4], the modulation of gene transcription, and also non-genomic actions. For example, some observations have been reported regarding TH actions at integrin αv/β3 in NTIS associated with cancer [39].

In conclusion, our data strongly support the presence of oxidative stress and NTIS in chronic kidney disease. Our findings highlight the potential adverse impact of reduced fT3 levels on ec-SOD activity and, consequently, endothelial function. We can thus speculate that if fT3 serves a protective role, it could offer substantial benefits in managing chronic kidney insufficiency, potentially delaying the need for dialysis treatment.

To reinforce these conclusions, further analysis should integrate our results with broader current research. Additionally, it is imperative to consider the practical implications of these findings for clinical management and patient care. Acknowledging the limitations of our study, it is recommended that future investigations delve deeper into the mechanisms underlying the relationship between fT3 levels, oxidative stress, and endothelial function in the context of CKD.

## Figures and Tables

**Table 1:** Metabolic parameters in the Acute Haemodialysis (AH) group and the Chronic Haemodialysis (CH) group.

		Glucose (mg/dL)	Creatinine (mg/dL)	Uric Acid (mg/dL)	Triglycerides (mg/dL)	Cholesterol (mg/dL)	LDL-C (mg/dL)	HDL-C (mg/dL)	Albumin (g/dL)	GOT (U/L)	GPT (U/L)	CRP (mg/L)
AH	Mean (SD)	103.5 (34.7)	6.81 (2.92)	7.04 (2.22)	193 (28)	115 (29.51)	56 (6)	25.50 (0.71)	27 (5.69)	14.67 (0.58)	20.10 (20.06)	37.25 (12.19)
CH	Mean (SD)	99.25 (34.14)	9.54 (2.23)	6.18 (1.20)	151 (14)	143.28 (37.15)	77 (5)	42.29 (14.01)	14.66 (14.29)	11.37 (4.6)	10.56 (6.08)	16.72 (7.16)
AH-CH	*p*-value	0.730	0.003 *	0.129	0.153	0.051	0.054	0.104	0.048 *	0.228	0.015 *	0.141

GOT = Glutamic-Oxaloacetic Transaminase; GPT = Glutamic-Pyruvic Transaminase. * indicates *p* < 0.05.

**Table 2:** Thyroid function and antioxidant measures in the two groups of patients studied. LAG = latency phase reported in seconds before the appearance of radicals, according to the method cited in the text, as a measure of total antioxidant capacity (TAC). The ec-SOD levels are reported in U/mL.

Group		TSH (μUI/mL)	fT4 (pg/mL)	fT3 (pg/mL)	LAG (s)	ec-SOD Basal 1 (Long)	ec-SOD Basal 2 (Short)	ec-SOD at 5 min	ec-SOD at 10 min	ec-SOD Final
AH	Mean (SD)	2.09 (1.52)	10.30 (3.7)	2.29 (0.91)	90 (23.91)	123.11 (19.8)	101.84 (22.72)	134.07 (32.95)	130.27 (33.97)	113.42 (28.15)
CH	Mean (SD)	2.08 (1.78)	9.38 (1.73)	2.30 (0.46)	68.48 (17.52)	247.61 (101.61)	231.711 (102)	276.88 (95.22)	275.18 (99.87)	226.74 (90.36)
AH-CH	*p*-value	0.993	0.311	0.952	0.006 *	0.011 *	0.001 *	<0.001 *	<0.001 *	<0.001 *

Basal 1 = basal fasting sample collected after a long interval (3 days after the last dialysis session); Basal 2 = basal fasting sample collected after a short period (2 days after the last dialysis session); * indicates *p* < 0.05.

**Table 3:** The ec-SOD activity before and after heparin in the two sub-groups, defined according to fT3 levels.

Group		ec-SOD Basal 1 (Long)	ec-SOD Basal 2 (Short)	ec-SOD at 5 min	ec-SOD at 10 min	ec-SOD Final
Low fT3	Mean (SD)	211.69 (88.34)	207.30 (86.86)	250.75 (93.80)	247.06 (95.82)	204.21 (90.64)
Normal fT3	Mean (SD)	296.45 (74.47)	261.62 (110.44)	316.02 (78.55)	312.04 (90.84)	243.61 (93.21)
Low-normal fT3	*p*-value	0.006 *	0.108	0.031 *	0.045 *	0.208

The ec-SOD levels are reported in U/mL. * indicates *p* < 0.05.

**Table 4:** Pearson correlation matrix between fT3 and ec-SOD at baseline and at the times considered.

Variables of Interest	fT3	ec-SOD_Long	ec-SOD_Short	ec-SOD_5min	ec-SOD_10min	ec-SOD_Final
fT3	1					
ec-SOD_long	0.443 ** (0.010)	1				
ec-SOD_short	0.251 (0.159)	0.802 *** (0.000)	1			
ec-SOD_5min	0.295 (0.096)	0.820 *** (0.000)	0.911 *** (0.000)	1		
ec-SOD_10min	0.294 (0.096)	0.811 *** (0.000)	0.936 *** (0.000)	0.975 *** (0.000)	1	
ec-SOD_final	0.115 (0.524)	0.664 *** (0.000)	0.686 *** (0.000)	0.730 *** (0.000)	0.717 *** (0.000)	1

Numbers represent correlation coefficients (r), *p*-values are given in parentheses. Asterisks are used to indicate the level of statistical significance (*p*-values in parentheses: ** = *p* < 0.01, *** = *p* < 0.001). The variables are analysed by pairwise correlation, so it is possible to read the coefficients and their *p*-values in the intersection of rows and columns. The ec-SOD levels are reported in U/mL. fT3 = free triiodothyronine; ec-SOD_long = basal fasting sample collected after a long interval; ec-SOD_short = basal fasting sample collected after a short period; ec-SOD_5min = activity values after heparin infusion at 5 min; ec-SOD_10min = activity values after heparin infusion at 10 min; ec-SOD_final = activity values at the end of treatment.

**Table 5:** Comparison of ec-SOD activity (U/mL) over time during and at the end of the dialysis vs. basal levels.

Group		ec-SOD at 5 min vs. Basal	ec-SOD at 10 min vs. Basal	ec-SOD at the End vs. Basal	Subgroup	ec-SOD at 5 min vs. Basal	ec-SOD at 10 min vs. Basal	ec-SOD at the End vs. Basal
AH	Mean (SD)	27.09 (11.83)	22.06 (11.61)	7.40 (20.84)				
CH	Mean (SD)	45.17 (34.13)	43.47 (34.84)	−4.97 (53.95)	Low fT3	43.45 (18.69)	39.76 (21.26)	−3.08 (87.09)
					Normal fT3	54.40 (58.01)	50.42 (48.52)	−18.02 (65.09)
AH-CH	*p*-value	0.011 *	0.078	0.928		0.232	0.391	0.217

* *p* < 0.05.

## Data Availability

All data are available in the paper. Crude data will be available on request due to restrictions (privacy and ethical).
